# C-754-08. Utilizing Single-Cell RNA Sequencing to Compare Hypopigmented vs Hyperpigmented Burn Scar for Cell-Specific Gene Targets

**DOI:** 10.1093/jbcr/irag033.036

**Published:** 2026-04-06

**Authors:** Jamie G Oh, Casey Meretta, Eriks Ziedins, Jeffrey W Shupp, Bonnie C Carney

**Affiliations:** The Burn Center at MedStar Washington Hospital Center, Washington, DC; The Burn Center at MedStar Washington Hospital Center, Washington, DC; Medstar Washington Hospital Center, Washington, DC; Medstar Washington Hospital Center, Washington, DC; The Burn Center at MedStar Washington Hospital Center, Washington, DC

## Abstract

**Introduction:**

Hypopigmentation is a common problem after burn injury – resulting in morbidities for patients. Studies show that hypopigmented scars contain melanocytes, but they lack normal function. The mechanism causing this dysfunction is unknown and no treatment exists. Bulk transcriptomics studies have failed to move the needle on dyschromic scar treatment. This study aims to explore differences in RNA expression through single cell RNA sequencing in hypo- compared to hyper-pigmented scars.

**Methods:**

Dyschromic scars from an established Red Duroc swine model were used for study. At day 70, a dermatome was used to obtain split-thickness scar containing epidermal and upper dermal layers. Tissue was split into hyper- and hypo-pigmented areas (n = 4 hyper, n = 4 hypo). Samples were enzymatically and mechanically digested to obtain single cells in each sample. Raw reads were filtered, low quality reads were removed, and clean reads were mapped onto a porcine reference transcriptome. UMAPs were created to identify cell clusters. Cell types were identified by comparing differential gene expression between cell clusters of known skin- and immune-cell specific genes.

Cell counts and proportions were compared utilizing chi-square analysis. Pseudo-bulk RNA sequencing, taking all cell types as a whole, was performed to compare hyperpigmented scar to hypopigmented scar to obtain lists of differentially expressed genes (DEGs).

**Results:**

Nine cell types were identified in the scar samples. The predominant cell type in both groups were keratinocytes followed by fibroblasts. Melanocytes made up the fewest count of cells. When comparing the proportion of cell types, there was a higher proportion of melanocytes in the hyperpigmented group when compared to the hypopigmented group (*p*-value <0.05).

Pseudo-bulk RNA sequencing comparing hyper- and hypo-pigmented samples revealed 13 DEGs between the groups. These genes were predominantly expressed in keratinocytes, with the exception of HSPA6, CRYAB, and CFD being expressed more strongly within fibroblasts. Among these genes, several have known implications on pigmentation including BNC2, HSPA6, and SOX5 (Table 1).

When comparing individual cell types, there were no DEGs between hyper- and hypo-melanocytes. Within fibroblasts there were 27 DEGs and in keratinocytes there were 5 DEGs between hyper- vs hypo-pigmented groups.

**Conclusions:**

Through the use of single cell RNA sequencing, several pigment related genes were revealed to be differentially expressed and may represent novel cell-specific targets to elucidate the mechanism behind melanocyte dysfunction in hypopigmented burn scar.

**Applicability of Research to Practice:**

Genes of interest revealed in this comparison may be novel targets for therapeutic agents to activate melanocyte function in hypopigmented burn scars. Importantly, these genes are not melanocyte-specific and therefore position paracrine signaling as a viable target for altering pigmentation in burn scar.

**Funding for the study:**

This work was funded in part by award number KL2TR001432 from the National Center for Advancing Translational Science (NCATS/NIH).

